# Alterations in empathic responding among women with posttraumatic stress disorder associated with childhood trauma

**DOI:** 10.1002/brb3.215

**Published:** 2014-03-13

**Authors:** Melissa Parlar, Paul Frewen, Anthony Nazarov, Carolina Oremus, Glenda MacQueen, Ruth Lanius, Margaret C McKinnon

**Affiliations:** 1 Department of Psychiatry and Behavioral Neurosciences, McMaster UniversityHamilton, Ontario, Canada; 2 Mood Disorders Program, St. Joseph's HealthcareHamilton, Ontario, Canada; 3 Department of Psychiatry, University of Western OntarioLondon, Ontario, Canada; 4 Department of Psychiatry, Hotchkiss Brain Institute, University of CalgaryCalgary, Alberta, Canada; 5 Kunin-Lunenfeld Applied Research Unit, Baycrest CentreToronto, Ontario, Canada

**Keywords:** Adult survivors of child abuse, empathy, posttraumatic, stress disorders

## Abstract

**Objective:**

Although studies increasingly point toward problems with social cognition among individuals with posttraumatic stress disorder (PTSD), few studies have assessed empathic responding. The aim of the current study was to investigate empathic responding in women with PTSD related to childhood trauma, and the contribution of parental bonding to empathic abilities in this sample.

**Methods:**

Participants with PTSD (*n* = 29) and sex- and age-matched healthy controls (*n* = 20) completed two self-report empathy measures, the Interpersonal Reactivity Index (IRI) and the Toronto Empathy Questionnaire (TEQ), and a self-report measure of attachment, the Parental Bonding Instrument (PBI).

**Results:**

Women with PTSD, relative to controls, reported significantly lower levels of empathic concern (*r* = 0.29) and perspective taking (*r* = 0.30), yet significantly higher levels of personal distress (*r* = 0.45) on the IRI. Women with PTSD also reported elevated scores on the TEQ (*η*^2^ = 0.13). Levels of paternal care on the PBI, rather than childhood trauma severity or PTSD symptom severity best predicted perspective taking scores on the IRI in the PTSD sample (*R*^2^ = 0.20).

**Conclusion:**

Women with PTSD associated with childhood trauma reported alterations among different domains of empathic functioning that may be related to low levels of paternal care.

## Introduction

Empathy is an essential part of social behavior. It allows us to understand others by inferring and sharing their feeling states in reference to ourselves (Decety and Moriguchi 2007) and is considered imperative to many forms of adaptive social interaction (Spinella 2005). Despite well-established evidence of impaired interpersonal functioning among individuals with posttraumatic stress disorder (PTSD) (Olatunji et al. 2007), to date little work has examined deficits in social cognitive functioning, including empathy, in this population (Nietlisbach et al. 2010; Sharp et al. 2012; Nazarov et al. 2013). Here, we investigate empathic responding in a sample of women with PTSD following repeated childhood trauma (including neglect, physical and/or emotional and/or sexual abuse).

Predominant theoretical models of empathy propose that it is multidimensional and integrative in nature, consisting of both cognitive (i.e., inferring the thoughts and intentions of others using intellectual processes, often referred to as mentalizing) and emotional (i.e., feeling the affect and pain of others) components (Davis 1983; Rankin et al. 2005). The neural network associated with empathic functioning supports this multidimensional model and includes cognitive (e.g., dorsolateral prefrontal cortex), memory (e.g., hippocampus; temporal poles; anterior and posterior cingulate), and affective systems (e.g., amygdala; orbitofrontal and medial frontal cortices) (Eslinger 1998; Farrow et al. 2001; Vollm et al. 2006; Shamay-Tsoory 2011). Individuals may show alterations in these neural networks following exposure to trauma, subsequently affecting the cognitive, memory, and affective processes requisite to empathic responding (Vasterling et al. 2002; Clark et al. 2003; Koso and Hansen 2006; Etkin and Wager 2007; Jelinek et al. 2008; Moores et al. 2008; Hayes et al. 2009; Moore 2009).

PTSD exerts negative effects on interpersonal functioning (Olatunji et al. 2007); these deficits may relate, in part, to the disruption of empathic responding, which is considered crucial to competent social interactions. For example, emotional numbing, a key symptom of PTSD, is associated with the disruption of interpersonal functioning when assessed via self-report measures (Beck et al. 2009) and may also disrupt one's ability to empathize with others. Moreover, there are additional consequences of repeated childhood trauma that may enhance risk for alterations in empathic functioning. For example, childhood trauma is often associated with disorganized or insecure attachment, which is suspected to hinder the development of mentalizing (i.e., the process of making sense of one's own and other's mental states) (Allen 2003) and the cerebral structures that support its development (Schore 2001; Allen and Fonagy 2002). Secure attachment, on the other hand, is thought to foster the development of mentalizing (Bogdan 2003). This is of importance as mentalizing is thought to comprise the cognitive component of empathy (Wagner et al. 2011). Moreover, in one recent study, children with recent histories of physical abuse, perpetrated by one or both parents, performed worse on a cognitive perspective-taking task (Flavell et al. 1968) compared to children without histories of abuse (Barahal et al. 1981). Further, a strong negative association exists between maternal care and alexithymia, suggesting that dysfunctional parent–infant relationships contribute to reduced awareness of one's own feelings. This is an important finding given that higher rates of alexithymia are associated with deficits in empathy (Teten et al. 2008) and that alexithymia contributes to dysfunction in interpersonal relationships (Feldmanhall et al. 2013).

To our knowledge, only one study has systematically examined empathic responding in adults with PTSD (Nietlisbach et al. 2010). Nietlisbach et al. (2010) found that, compared to healthy controls, participants with a history of PTSD reported significantly higher levels of personal distress as assessed by the Interpersonal Reactivity Index (IRI) (Davis 1980, 1983), a commonly used self-report measure of empathic responding. Nonetheless, this was a highly mixed sample, where more than half were subsyndromal at the time of testing, and the types of traumatic events experienced were heterogeneous (i.e., 81% experienced a sexual assault, whereas 19% experienced an accidental trauma or natural disaster). Given that the psychiatric sequelae and physiological response associated with repeated developmental trauma and single-incident trauma differ (Green et al. 2000; van der Kolk et al. 2005; Nietlisbach et al. 2010), for the present study, we only included individuals who met full diagnostic criteria for PTSD related to childhood trauma at the time of testing.

Empathic responding has also been studied in patients with a diagnosis of borderline personality disorder (BPD). These findings may be of particular relevance here as there is a high rate of co-occurrence of PTSD and BPD with significant overlap in both phenomelogical aspects (e.g., affect dysregulation, dissociation) and the shared rates of exposure to adverse events (Pagura et al. 2010). In one study, the IRI was used to assess empathic functioning in a sample of women with BPD; women with BPD exhibited higher levels of personal distress and higher levels of empathic concern, as compared to healthy controls (Guttman and Laporte 2000).

### Aims of the study

We investigated empathic abilities in 29 women with PTSD associated with childhood trauma, as compared to 20 healthy women. In addition, due to role of parental bonding in social functioning and its disruption in individuals exposed to childhood trauma (Rikhye et al. 2008), we examined the predictive role that parental bonding may play in empathic abilities. Given the alterations in cognitive and affective processes seen in PTSD related to childhood trauma, we hypothesized that our patient sample would show alterations in the cognitive and affective components of empathic responding, as compared to controls.

## Materials and Methods

### Subjects

Forty-nine women participated in this study. There were two groups of participants: 29 women who met DSM-IV-TR diagnostic criteria for a primary diagnosis of current chronic PTSD due to a history of childhood trauma (PTSD group; mean age 42.5 [SD = 12.2] years), and 20 age- and sex-matched healthy controls (HC group; mean age 35.8 [SD = 13.2] years). Women with PTSD were recruited from London Health Sciences Centre (LHSC) in London, Ontario, Canada. Age-matched psychologically healthy women were recruited from St. Joseph's Healthcare Hamilton, in Hamilton, Ontario and LHSC.

Participants with a history of neurological disease, traumatic brain injury, and/or head injury with loss of consciousness (lasting more than 60 sec), substance abuse in the last 6 months, current or lifetime history of substance dependence, and/or current or prior history of untreated significant medical illness were excluded. In addition, women with PTSD were excluded if they had ever been diagnosed with bipolar disorder or a psychotic disorder and women in the psychologically healthy group were excluded if they had ever received psychotherapy. All participants provided written informed consent. The study was approved by the local Research Ethics Boards and was performed in accordance with the ethical standards laid down by the 1964 Declaration of Helsinki.

### Clinical assessments

PTSD diagnostic status and symptom severity was assessed using the Clinician-Administered PTSD Scale (CAPS) (Blake et al. 1995) and history of moderate-to-severe childhood trauma was confirmed for PTSD subjects by retrospective self-report using the Childhood Trauma Questionnaire (CTQ) (Bernstein et al. 2003). Depressive symptom severity was measured with the Beck Depression Inventory (BDI) (Beck et al. 1996). The CAPS and CTQ were also administered to the group of psychologically healthy women to rule out the presence of any PTSD-related symptoms and lifetime trauma history. In addition, the Structured Clinical Interview for DSM-IV-TR AXIS I Disorders (SCID-I) (First et al. 1996) was administered to identify comorbid Axis I conditions in the sample with PTSD and to rule out the presence of any current or past Axis I conditions in the control group. Demographic and clinical characteristics of the study sample are reported in Table 1.

**Table 1:** Demographic and clinical characteristics of the study sample

	PTSD (*n* = 29)	Controls (*n* = 20)
Demographics		
Age, years, mean (SD)	42.5 (12.2)	35.8 (13.2)
Years of education, mean (SD)	13.7 (2.4)	16.2 (2.6)^1^
Caucasian	93%	65%
Married/common-law/engaged	28%	45%
Separated/divorced	28%	20%
Single	34%	30%
Have children	45%	40%
Severity of PTSD symptoms		
CAPS, mean (SD), range	79.9 (15.5), 55–118	0.5 (1.6), 0–7^1^
Parental Bonding Instrument, mean (SD)		
Paternal care	11.22 (8.09)	30.93 (5.37)^1^
Paternal overprotection	17.19 (9.13)	10.53 (6.46)^1^
Maternal care	14.56 (8.82)	30.00 (6.92)^1^
Maternal overprotection	16.63 (9.38)	9.93 (5.82)^1^
Childhood Trauma Questionnaire, mean (SD)^2^, %ile equivalent of mean^3^		
Total	76.6 (20.46)	29.7 (4.23)^1^
Emotional abuse	18.9 (4.8), 90–95th	6.2 (1.6)^1^, 40th
Physical abuse	12.6 (5.7), 90–95th	5.5 (1.1)^1^, 50th
Sexual abuse	15.5 (7.3), 90–95th	5.0 (0.0)^1^, 70th
Emotional neglect	18.0 (4.6), 90th	7.0 (1.9)^1^, 30th
Physical neglect	11.7 (5.4), 95th	6.0 (1.5)^1^, 70th

	Past	Current
Comorbid axis I conditions in the PTSD sample		
Alcohol dependence	1	0
Substance abuse	1	0
Major depressive disorder	16	11
Panic disorder w/wo agoraphobia	8	3
Agoraphobia	4	5
Social phobia	1	4
Specific phobia	1	3
Obsessive compulsive disorder	0	2
Somatization disorder	3	8
Dissociative identity disorder	1	4
Anorexia nervosa	4	2
Bulimia nervosa	2	1

CAPS, Clinician-Administered PTSD Scale; PTSD, posttraumatic stress disorder; SD, standard deviation.

1 Significant group effect (*P* < 0.05).

2 Minimum score for each CTQ scale is 5.

3 Percentiles relative to the normative population of female health management organization members (*N* = 1187) described in Bernstein and Fink (1998, Table 4.5).

### Assessments of empathic responding

The IRI (Davis 1980, 1983) is a 28-item, self-report questionnaire based on the multidimensional models of empathy consisting of four, 7-item subscales, designed to tap different cognitive and emotional components of empathy. Specifically, the perspective taking and fantasy subscales measure the cognitive aspects of empathy, whereas the empathic concern and personal distress subscales measure the emotional aspects of empathy. The perspective taking subscale assesses the ability to take on the psychological point of view of others, allowing one to anticipate the behavior and reactions of others (e.g., *I sometimes find it difficult to see things from the “other guy's” point of view*). This subscale is associated with emotional sensitivity (Cliffordson 2002). The fantasy subscale assesses the tendency to imagine oneself experiencing the feelings and behaviors of fictitious characters in books, movies, and plays (e.g., *after seeing a play or movie, I have felt as though I were one of the characters*) and may be related to imagination (Baron-Cohen and Wheelwright 2004), general verbal skills, and the ability to engage others in social interaction (Cliffordson 2002). The empathic concern subscale measures the tendency to experience feelings of sympathy and concern for unfortunate others (e.g., *I often have tender, concerned feelings for people less fortunate than me*). This subscale is also reflective of an ability to receive and understand verbal communication (Cliffordson 2002) and individuals scoring high in empathic concern tend to have good general knowledge regarding the norms of appropriate social behavior (Riggio 1986; Riggio and Tucker 1989). Finally, the personal distress subscale assesses personal anxiety and discomfort experienced in emotional social settings (e.g., *being in a tense emotional situation scares me*). This subscale is thought to measure self-control (Baron-Cohen and Wheelwright 2004) and is positively related to neuroticism (Cliffordson 2001) and social sensitivity, and negatively related to emotional and social control (Cliffordson 2002). Items are rated on a scale ranging from 0 (does not describe me well) to 4 (describes me very well). The IRI has good test–retest reliability, good internal consistency (with indices ranging from 0.70 to 0.78), and adequate levels of convergence with other measures of empathy (Davis 1980, 1983; Christopher et al. 1993; Blake et al. 1995).

The Toronto Empathy Questionnaire (TEQ) (Spreng et al. 2009) is a 16-item self-report questionnaire that measures a broad range of empathic responses, emphasizing the emotional components of empathy. The items used in the TEQ appear to tap similar constructs as those represented by the empathic concern subscale of the IRI. Items are rated on a scale ranging from 0 (never) to 4 (always). A high score on the TEQ represents high self-reported levels of affective insight into the feeling states of others (Spreng et al. 2009). The TEQ has shown good internal consistency (Cronbach's α = 0.85), high test–retest reliability, and strong convergent validity (Spreng et al. 2009).

### Assessment of parental bonding during childhood

The Parental Bonding Instrument (PBI) (Parker et al. 1979) is a 25-item self-report questionnaire designed to assess parental bonding through two perceived parenting styles of the mother and father during the first 16 years of life: (1) care (e.g., *my mother/father was affectionate to me*) and (2) overprotection (e.g., *my mother/father tried to control everything I did*). High care and low overprotection are considered optimal, whereas low care and high overprotection are considered least optimal. Each item is scored on a 4-point scale ranging from 1 (very like) to 4 (very unlike) and assessed separately for mother and father. Scores on the PBI demonstrate good concordance with sibling ratings (Parker 1990) and do not merely reflect current depressed mood state (Duggan et al. 1998). The PBI shows high test–retest reliability over months, and moderate consistency over extended periods of up to 10 years (Parker 1990).

### Statistical analyses

Due to non-normality of the IRI subscales (Shapiro-Wilk, *P* < 0.05), these scores were log transformed in order to perform a parametric analysis. The log transformation, however, did not result in a normal distribution of scores among all of the IRI subscales (Shapiro-Wilk, *P* < 0.05). Therefore, the group differences on these subscales were analyzed using the nonparametric Mann–Whitney *U*-test (using the nonlog-transformed scores). In order to examine group differences on the normally distributed TEQ scores (Shapiro-Wilk, *P* > 0.05), these data were analyzed using a univariate analysis of variance (ANOVA), treating PTSD and control groups as fixed variables and the TEQ total score as the dependent variable. Estimated effect sizes were estimated by *r* for the Mann–Whitney *U*-test and by partial eta-square (*η*^2^) for the ANOVA.

Follow-up multiple linear regression analyses using stepwise entry were conducted within the group with PTSD only, setting the empathy scores that differed significantly from controls as the dependent variable and including the following predictor variables: CTQ total scores, CAPS total scores (from previous month), PBI paternal care scores, PBI paternal overprotection scores, PBI maternal care scores, PBI maternal overprotection scores, and years of education. Given the high prevalence of comorbid major depressive disorder (MDD) among our sample with PTSD (i.e., 11/29 current MDD; 16/29 past MDD), supplementary correlation analyses were conducted to determine if there is an association between scores on the BDI and empathy measures. Pearson's *r* or Spearman rho (*ρ*) values were reported, depending on results from the Shapiro–Wilk test of normality. Alpha was set at 0.05 for all analyses.

## Results

### Group comparisons for responses on the empathy measures

Table 2 reports the means, standard deviations, and group comparisons for IRI and TEQ scores. Women with PTSD reported lower levels of perspective taking (*U* = 187, *z* = −2.10, *P* = 0.035, *r* = 0.30) and empathic concern (*U* = 192, *z* = −2.00, *P* = 0.045, *r* = 0.29), and higher levels of personal distress (*U* = 137, *z* = −3.12, *P* = 0.002, *r* = 0.45) on the IRI relative to controls. There were no significant group differences between mean scores on the fantasy subscale.

**Table 2:** Between group differences on empathy measures

	PTSD	Controls	Test statistic	Effect size
Interpersonal Reactivity Index				
Perspective taking	16.8 (5.2)	20.3 (4.9)	*U* = 187*	0.30^*r*^
Personal distress	15.2 (5.8)	9.8 (4.5)	*U* = 137**	0.45^*r*^
Fantasy	13.9 (5.3)	13.9 (5.6)	*U* = 282	0.02^*r*^
Empathic concern	20.3 (5.0)	23.1 (3.9)	*U* = 192*	0.29^*r*^
Toronto Empathy Questionnaire	66.5 (13.1)	57.5 (8.8)	*F* (1, 47) = 7.13**	0.13^*η*^^2^

Values are *n* or mean (SD). Values denoted by *r* indicate a Mann–Whitney *U* effect size estimate. Values denoted by *η*^2^ indicate an ANOVA effect size (partial eta-squared).

* *P* < 0.05,

** *P* < 0.01.

Relative to controls, the PTSD group reported higher levels of empathic responding as assessed by the TEQ, *F*(1, 47) = 7.13, *η*^2^ = 0.13.

### Parental bonding, current PTSD symptom severity, childhood trauma severity, and years of education as predictors of empathic responding

PBI paternal care was the best predictor of IRI perspective taking, accounting for 20% of the variance (*R*^2^ = 0.197; adjusted *R*^2^ = 0.164, *F*(7, 25) = 5.893, *P* = 0.023). Only PBI paternal care significantly predicted perspective taking (*t*(25) = 2.43, *b* = 0.293; *P* = 0.023). None of the independent variables entered into the regression models significantly predicted IRI personal distress, IRI empathic concern, or TEQ scores. Therefore, PTSD symptom severity, as assessed by the CAPS, did not predict scores on any of the empathy subscales. To explore if any specific criteria of PTSD symptomatology, rather than total symptom severity, was related to empathy, a correlation analysis was performed to determine if the scores from CAPS criterion A, B, C, or Associated Features (from previous month) were associated with empathy scores in the group with PTSD. The only significant correlation that emerged was between criterion D (hyperarousal) and TEQ scores (*ρ* = 0.41, *P* = 0.029).

### Supplementary analyses

BDI scores indexing severity of potential comorbid depressive symptoms were not significantly correlated with IRI perspective taking (*ρ* = 0.20, *P* = 0.309), IRI empathic concern (*ρ* = 0.33, *P* = 0.082), IRI personal distress (*r* = 0.18, *P* = 0.356), IRI fantasy (*r* = 0.27, *P* = 0.158), or TEQ total (*ρ* = 0.22, *P* = 0.261).

The distribution of empathy scores among the group with PTSD is of further interest as it may be expected that some individuals with PTSD have impaired empathy, while others may have exaggerated empathy. The distribution of empathy scores among the sample with PTSD, as represented by the standard score of the skewness, was as follows: IRI fantasy: 0.77, IRI perspective taking: −1.64, IRI empathic concern: −2.00, IRI personal distress: 0.82, and TEQ: −2.35.

## Discussion

To our knowledge, this study is the first to reveal alterations in empathic responding among women with PTSD related to childhood trauma. Although women with PTSD reported a reduced ability to identify the social cognitive perspective of others (IRI perspective taking) and reduced feelings of care and concern in response to another's emotional experience (IRI empathic concern), their levels of personal distress in response to learning of others' negative experiences (IRI personal distress) were higher than those reported by matched controls. Of the empathy subscales that differed significantly between groups, the only one that was predicted by clinical variables was IRI perspective taking. Specifically, higher levels of self-reported PBI parental care predicted higher levels of self-reported perspective taking ability among women with PTSD.

The finding of reduced perspective taking ability in the PTSD group is novel in the literature and suggests deficits in cognitive empathic abilities among women with PTSD associated with childhood trauma. Although previous studies, including work in our own laboratory (Cusi et al. 2011), indicate that participants with MDD report reduced levels of perspective taking, this pattern did not emerge in Nietlisbach et al.'s (2010) study, where levels of perspective taking did not differ between participants with PTSD and controls. Critically, Nietlisbach et al. studied a group with PTSD that differed extensively from our group of participants with PTSD in terms of symptom severity, type of trauma exposure, and sex of participants, with half of the sample consisting of males. The present finding that women with a history of developmental trauma exposure showed reduced levels of perspective taking, an ability thought central to Theory of Mind (ToM), is in line with our earlier report that this sample shows alterations in mental state identification and in the perception of kinship interactions (Nazarov et al. 2013). Further work will be required to understand the relation between cognitive functioning (e.g., reduced working memory; poor executive functioning) and perspective taking in individuals with PTSD, as perspective taking is thought to rely on cognitive resources.

Although women with PTSD showed reduced cognitive empathy (i.e., IRI perspective taking), they scored higher than controls on the emotional IRI subscale of personal distress. The personal distress subscale, which assesses anxiety and discomfort experienced in emotional social settings, is associated with social dysfunction, fearfulness, emotional vulnerability, shyness, uncertainty, and anxiety (Davis 1983). Heightened levels of personal distress in women with PTSD in the present sample are consistent with the results reported by Nietlisbach et al. (2010). Notably, women with BPD also report higher levels of personal distress than controls (Guttman and Laporte 2000). Moreover, complementary results were observed in the present study with the TEQ, also considered to be an emotion-based measure of empathy.

Our results provide preliminary evidence that women with PTSD following a history of childhood trauma report less feelings of care and concern in response to other's emotional experiences, as assessed by the empathic concern subscale on the IRI. A reduction in empathic concern was also observed in individuals with MDD (Cusi et al. 2011) and may reflect the preoccupation with the self and negative ruminations often seen in those with depression (Beck 1967; Raes et al. 2006), rather than disinterest in another's well being. These results are in contrast with empathic responding in women with BPD who report increases in empathic concern (Guttman and Laporte 2000), which may be reflective of the “especially empathic” pattern often noted in BPD. Interestingly, however, women did show preserved function on the fantasy subscale of the IRI, a cognitive facet of empathic responding, indicating that cognitive empathic abilities are not globally disrupted in PTSD and supporting the observation that individuals with PTSD are just as likely to help others as healthy controls (Stotland 1978). An important conclusion is therefore that empathic responding is *altered*, rather than *reduced or impaired*, in individuals with PTSD. Our results support Davis's (1983) model of empathy as a multidimensional construct, consisting of both emotional and cognitive components.

An important characteristic of our patient sample is that the diagnosis of PTSD is associated with a history of repeated childhood trauma, rather than single-incident adult trauma. Among this sample, higher levels of paternal care on the PBI were predictive of higher scores on the perspective taking subscale of the IRI. In contrast, neither severity of childhood trauma, severity of current PTSD symptoms, nor years of education predicted empathic abilities, indicating that attachment during childhood, rather than trauma-related symptomatology or education history, may have the strongest impact on empathic functioning. Given that women with PTSD in our sample were repeatedly abused and/or neglected during childhood, it is possible that the perpetrator was the father in many of these cases, which may explain why levels of paternal care, but not maternal care, predicted empathic responding. Nonetheless, this finding highlights the need to focus on the role of the father, rather than only the more often-studied role of the mother, in the development of empathy and more broadly, social cognition. Frewen et al. (2013) found that higher levels of paternal emotional availability but not maternal emotional availability (as assessed by the Childhood Attachment and Relational Trauma Screen; Frewen et al. 2013) were related to less trait negative effect in childhood in a sample of undergraduate students.

Other work also supports the notion that altered parental bonding contributes to aberrant development of empathy. For example, individuals who have experienced attachment trauma are more prone to hyperarousal (Schore 2002) which typically reduces one's ability to mentalize. Given that empathy is a component of mentalizing, this reduced capacity for mentalizing is likely reflected in the lowered levels of perspective taking ability seen in our sample, stemming from lower levels of perceived care offered by parents (as indicated in the PBI). When considering the experiences of a child growing up in a hostile environment where his or her caregiver is the perpetrator, it seems reasonable to suspect the development of the child's perspective-taking abilities would be hindered. Indeed, past research has suggested that low levels of empathy are associated with the presence of aggressive and bullying behaviors (Castano 2012). Thus, not only could potentially low levels of empathy among parents/perpetrators be associated with the maltreatment of one's child, but this environment may provide poor modeling for the child, subsequently affecting development of empathy. Further, it is likely that for many children who are victims of maltreatment by their caregivers, it may simply be too frightening and aversive to take on the perspective of their parents, which may ultimately generalize to interpersonal situations with nonperpetrators. Critically, identification of mechanisms underlying the intergenerational transmission of the deleterious effects of trauma exposure (e.g., increased risk of subsequent abusive behavior by offspring) will be central to intervention efforts (e.g., programs aimed at enhancing interpersonal sensitivity) aimed at reducing these effects.

There are several limitations to the present study that should be addressed in future research. First, our measures, while well-validated, consisted entirely of retrospective self-report questionnaires. Future studies should include behavioral/non-self-report measures of empathy and use prospective designs. In addition, because our sample consisted of women with histories of complex trauma, the present results cannot be generalized to men or to individuals who have experienced traumatic events only in adulthood.

The current results suggest that empathy is not globally disrupted in PTSD stemming from childhood trauma, but that instead only select aspects (i.e., perspective taking, personal distress, empathic concern) are altered, while others (i.e., fantasy) remain spared. Furthermore, self-reported levels of parental care were more predictive of perspective-taking abilities than were severity of childhood trauma or current PTSD symptom severity. Enhanced knowledge in the field of social cognitive functioning in PTSD may assist the development of strategies to improve social functioning with an aim of increasing the capacity to utilize social support. This is an important goal given that a lack of social support presents as the strongest risk for the maintenance of PTSD symptomatology (Brewin et al. 2000).
